# The Footprint of Exosomes in the Radiation-Induced Bystander Effects

**DOI:** 10.3390/bioengineering9060243

**Published:** 2022-05-31

**Authors:** Safura Jokar, Inês A. Marques, Saeedeh Khazaei, Tania Martins-Marques, Henrique Girao, Mafalda Laranjo, Maria Filomena Botelho

**Affiliations:** 1 Department of Nuclear Pharmacy, Faculty of Pharmacy, Tehran University of Medical Sciences, Tehran P94V+927, Iran; jokar.safura@gmail.com; 2 Institute of Biophysics, Faculty of Medicine, University of Coimbra, 3000-548 Coimbra, Portugal; ines.marques@student.uc.pt (I.A.M.); mafaldalaranjo@gmail.com (M.L.); 3 Coimbra Institute for Clinical and Biomedical Research (iCBR), Faculty of Medicine, University of Coimbra, 3000-548 Coimbra, Portugal; tania.m.marques@fmed.uc.pt (T.M.-M.); hmgirao@fmed.uc.pt (H.G.); 4 Center for Innovative Biomedicine and Biotechnology (CIBB), University of Coimbra, 3000-548 Coimbra, Portugal; 5 Centre of Investigation in Environment, Genetics and Oncobiology (CIMAGO), Faculty of Medicine, University of Coimbra, 3000-548 Coimbra, Portugal; 6 Faculty of Pharmacy, University of Coimbra, 3000-548 Coimbra, Portugal; 7 Department of Pharmaceutical Biomaterials, Faculty of Pharmacy, Tehran University of Medical Sciences, Tehran P94V+927, Iran; saeede.khazaei@gmail.com; 8 Clinical and Academic Centre of Coimbra (CACC), 3004-561 Coimbra, Portugal

**Keywords:** radiation therapy, exosome, bystander effects, radioresistance, cancer

## Abstract

Radiation therapy is widely used as the primary treatment option for several cancer types. However, radiation therapy is a nonspecific method and associated with significant challenges such as radioresistance and non-targeted effects. The radiation-induced non-targeted effects on nonirradiated cells nearby are known as bystander effects, while effects far from the ionising radiation-exposed cells are known as abscopal effects. These effects are presented as a consequence of intercellular communications. Therefore, a better understanding of the involved intercellular signals may bring promising new strategies for radiation risk assessment and potential targets for developing novel radiotherapy strategies. Recent studies indicate that radiation-derived extracellular vesicles, particularly exosomes, play a vital role in intercellular communications and may result in radioresistance and non-targeted effects. This review describes exosome biology, intercellular interactions, and response to different environmental stressors and diseases, and focuses on their role as functional mediators in inducing radiation-induced bystander effect (RIBE).

## 1. Introduction

Cancer is a major health issue in the world, accounting for about 10.0 million deaths worldwide in 2020, as estimated by International Agency for Research on Cancer (IARC). Due to the rapid growth and aging of the population and the increasing prevalence of high-risk factors, it is expected that the number of cancer-affected patients will reach more than 28.4 million cases worldwide by 2040 [1,2]. Radiation therapy (RT) is one of the comprehensive and highly cost-effective modalities for cancer patients, accounting for only 5% of the total cost of cancer therapy [3,4]. Electromagnetic radiations (X-rays or gamma rays) are types of radiation used in RT, with X-rays being generated through linear accelerators (LINAC), while gamma rays are emitted during radioactive nucleus decay (cobalt-60 at gamma-knife). These radiations are considered low linear energy transfer (LET) [3]. The X-rays from LINACs are widely applied in more than 50% of cancer patients for curative and palliative purposes, separately or combined with other treatment modalities, including surgery, chemotherapy, and immunotherapy [5,6,7]. Since such modality non-specifically targets the tumour tissues by ionising radiation beams, healthy tissue could also be damaged in the RT process and lead to cytotoxicity and the emergence of metastasis or even long-term new tumours [8]. Valuable progress has been made on radiation delivery strategies, leading to the increasing use of particle radiation, namely electron, proton, and neutron beams and heavy ions. Electron beams are the most commonly used to deliver radiation to the tumour without deep penetration into tissues, but the use of other particles, such as heavy ions and especially protons, has also been increasing. The proton beam is the most recent and can offer a better dose deposition on the tumour site, minimizing surrounding tissues. The use of protons and heavy ions have been increasingly associated with the growing number of particle radiotherapy centres. These types of particle radiation have high LET than photon-based radiation and can have higher biological effectiveness [3]. This is a huge advance for clinical cancer therapy considering the capacity of heavy charged particles in depositing most of their energy at tumour site and lower deposition in surrounding normal tissues. RT using low LET radiation, such as photon, induced cell death mostly by free radical production and water radiolysis while high LET radiation acts mostly by lethal damage in DNA, with a low possibility of DNA repair [9]. This damage was evidenced both in direct irradiated cells or tissues as well as in distant non-irradiated, for different types of radiation and tissues [10]. 

Still, nontargeted effects of radiation on the neighbouring and distant tumour tissues are a relevant topic in radiation oncology, radiobiology, and radioprotection [8]. 

The non-targeted effects could be classified into three types—bystander, abscopal, and cohort effect—based on the interplay among irradiated and nonirradiated cells, types of cells, and the distance from the irradiated local. The United Nations Scientific Committee on the Effects of Atomic Radiation (UNSCEAR) classifies radiation-induced bystander effects (RIBEs) as a local radiobiological effect transmitted only over a few millimeters or cell diameters from irradiated cells to neighbouring nonirradiated cells. Abscopal effects refer to effects extended from treated tissue volume to distant locals, usually associated with immunogenic response. There are no topographical limit definitions to well distinguish between RIBE and abscopal effects, but RIBE is generally considered local communicative effects at the primary site while abscopal is a long-distance, out of irradiated volume, and systemic effects responsible for the effects at secondary metastatic lesions. The less known effects, the cohort effects, are limited to millimeters inside a target irradiated volume and define the action between cells heterogeneously irradiated with high and low-dose and how their interactions could affect the whole tissue volume response [11]. RIBE encompasses complex biological processes mediated by intercellular signals from irradiated cells through secretion of soluble factors, gap junction signalling, or networks involving inflammatory cells of the microenvironment [11,12]. Consequences of signalling pathways, gene mutations, chromosomal aberrations, DNA damage, apoptosis, autophagy, and inflammatory response could lead to carcinogenesis and metastasisation [13,14,15,16,17].

**Figure 1:**
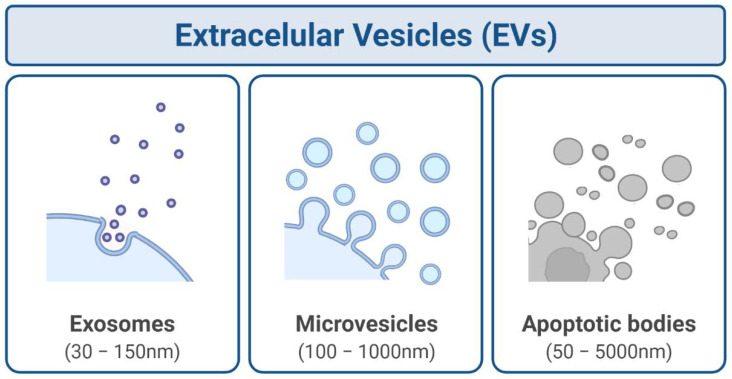
Extracellular vesicles (EVs) can be classified into exosomes, microvesicles, and apoptotic bodies, according to their size and biogenesis mechanism. Created with BioRender.com.

Extracellular vesicles are functional and critical mediators of the tumour microenvironment, progression, metastasis, and radiotherapeutic responses. Extracellular vesicles are means of intercellular communication between the irradiated and nonirradiated cells and induction of RIBEs [8,11,18,19,20,21,22,23]. In general, nano-sized phospholipid vesicles released by the cells to the extracellular microenvironment can be classified into three major classes. Exosomes (30–150 nm), microvesicles (100–1000 nm), and apoptotic bodies (50–5000 nm) are represented in Figure 1, according to different subcellular origins, composition, and size distributions [24,25]. Due to the lack of specific markers and techniques to unequivocally purify each subset of vesicles, the International Society of Extracellular Vesicles (ISEV) currently recommends using the generic term extracellular vesicles [26]. Once putatively, every cell type can release extracellular vesicles, they can be found in virtually all biological fluids, including the blood, saliva, milk, amniotic fluid, cerebrospinal fluid, and urine [27]. As one of the primary cell-derived nanovesicle mediators, Peter Wolf first described exosomes in 1967 [28]. These secreted vesicles are constituted by complex bioactive components, including proteins, lipids, and nucleic acids [20,29,30,31,32].

Extracellular vesicles’ importance in regulating immune response, disease progression, and cell-cell communication explains the significant research interest in the recent decade [33,34,35,36]. The molecular profile of these nanovesicles changes with the pathophysiological state of parental tumour cells [18,37]. Additionally, the small size of extracellular vesicles enables them to escape from phagocytosis by blood cells easily. Cross biological barriers facilitate extravasation from blood vessels and subsequent diffusion into tumour cells, which can be exploited for therapeutic purposes. In agreement, extracellular vesicle-based transporters have been used to deliver small molecules, such as paclitaxel, doxorubicin, imatinib, curcumin, antifungal drugs, and analgesics microRNA, and siRNA [38,39,40,41,42,43]. They could also be considered promising diagnostic biomarkers of cancer progression because of their availability in body fluids and their potential involvement in all stages of cancer [19,44,45]. 

Extracellular vesicle features, such as molecular cargos, secretion level, and potential binding affinity to recipient cells, primarily depend on the origin and the type of cell, and its maturation state. Still, these properties could be altered in response to pathological conditions, such as environmental stressors and during disease progression [29,46,47]. As one stressful condition, radiation may affect the abundance and composition of extracellular vesicles released by irradiated cells. Consequently, altered cell-cell communication may contribute to cancer progression, radioresistance, and radiation-associated secondary tumours (non-targeted effects) [29,32,48,49]. 

Therefore, this manuscript revises the extracellular vesicles’ biogenesis, intercellular interactions, response to different environmental stressors and diseases, and role in cancer progression and radioresistance. We will also specifically focus on the role of extracellular vesicles as functional mediators in the induction of radiation-induced bystander effect (RIBE).

## 2. Exosomes

Exosomes present overlapping properties with other extracellular vesicles. However, they have particular biogenesis, morphology, cargo molecules, and biological functions. Exosome biogenesis is more complex than secreted microvesicles, which are generated via “pinching off” or “budding” from the cell surface membranes [19].

**Figure 2:**
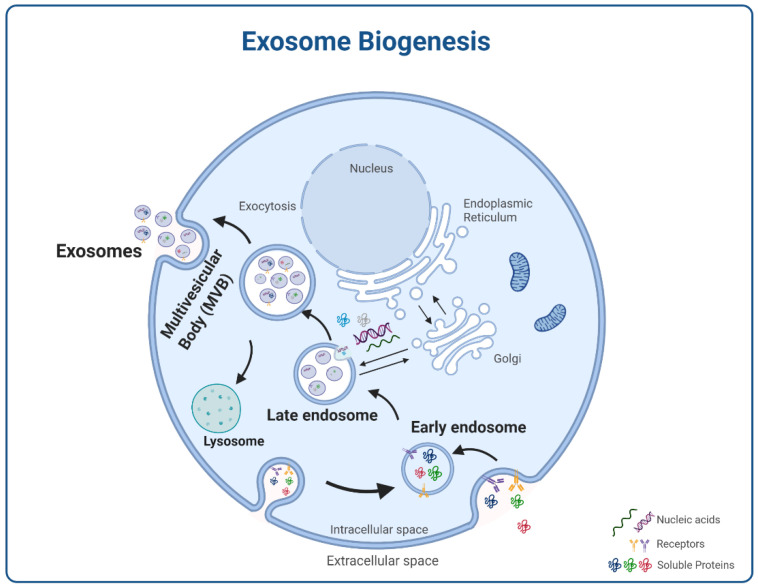
Exosome biogenesis comprises three sequential steps, starting from the invagination of the plasma membrane to form endocytic vesicles (that fuse to form early endosomes) in the intracellular space, followed by their maturation and additional cargo incorporation (nucleic acids, receptors, soluble proteins, etc.) giving rise to multivesicular bodies (MVBs). Lastly, these MVBs could either fuse with lysosomes for degradation or move to merge with the plasma membrane and release their vesicular content, the exosomes, to the extracellular space. Created with BioRender.com.

As shown in Figure 2, exosome biogenesis starts with plasma membrane endocytosis forming an endosome. After endosomes maturation into multivesicular bodies (MVBs), invaginations or inward buddings form the intraluminal vesicles that, after fusion with the plasma membrane, give rise to secreted exosomes [27,37].

Cells regulate exosome biogenesis and incorporation of molecular cargo through mechanisms dependent or independent of the endosomal sorting complex required for transport (ESCRT) [27,37]. The ESCRT-dependent pathway is the most common mechanism [50]. This complex encompasses four soluble multiprotein complexes, including ESCRT-0, ESCRT-I, ESCRT-II, and ESCRT-III. First, ubiquitinated cargo in specific microdomains within early endosomes can be recognised by the ESCRT-0 complex by its ubiquitin-binding subunits, including Hrs, STAM, and Eps15 proteins [51]. Then, Hrs interacts with Tsg101, a ubiquitin-binding subunit of the ESCRT-I complex, which activates the ESCRT-II complex to further recruit and assist oligomerisation and formation of the ESCRT-III complex that also involves the action of AIP1 (also known as Alix) [52]. These interactions induce deformation in the MVBs membrane for inward budding and sorting of cargo proteins into the invaginations. Recruitment of deubiquitinating enzymes by the ESCRT-III complex is also required to deubiquitinate cargo proteins before exosome loading. Finally, inward buddings are separated by the ESCRT-III complex from the MVBs membrane [53,54]. The machinery involved in cargo sorting into exosomes is still unclear. Identifying Alix and Tsg101 (ESCRT complex subunits) in proteomic analyses of exosomes provides solid evidence for ESCRT-dependent biogenesis [55]. Importantly, ubiquitin was demonstrated to act as a critical signal sorting of both soluble and membrane proteins into exosomes [56].

The ESCRT-independent pathway depends on the sorted molecular cargo within the donor cells. In this pathway, ceramide, formed by enzymatic cleavage of sphingomyelin by the action of neutral sphingomyelinase (nSMase)-2, participates in exosomal secretion by glial cells [57]. While Tsg101 or Alix proteins have no role in sorting proteolipid protein (PLP) into the oligodendroglial cells-derived exosomes, ceramide is required [57]. Other lipids, such as sphingosine-1-phosphate, have also been implicated in facilitating membrane invagination. Tetraspanin-enriched lipid microdomains are major regulators of ESCRT-independent exosome formation, being involved in the bioactive molecules loading into exosomes [57,58,59]. For example, CD9-enriched lipid microdomains contribute to the MHC-II sorting into exosomes via its incorporation [60].

More recently, a lysosome-associated membrane protein 2 isoform A (LAMP2A)-dependent mechanism was described as mediating the selective sorting of proteins into exosomes, particularly those containing KFERQ-like aminoacid sequences [61]. This mechanism was independent of the ESCRT machinery but relies on HSC70, CD63, Alix, Syntenin-1, Rab31, and ceramides.

### 2.1. Molecular Cargos

Numerous studies have confirmed that exosomes comprise specific molecular compositions compared with other extracellular vesicles, including a random set of cell debris components. Compositions are dependent on the donor cells’ type and maturation state, but environmental conditions can also affect contents [47]. Exosomes contain various proteins, nucleic acids, and lipids with unique biological functions. Currently, they are being systematised in the exosome database of ExoCarta [37]. 

The protein composition of exosomes is complex. Due to endosomal origin, exosomes carry proteins involved in the MVBs formation, such as Alix and TSG101. They include proteins involved in membrane fusion, such as RAB GTPases, Annexins, and Flotillins, and endosomal or membrane lipid microdomains associated with proteins, such as integrins, CD63, CD9, CD53, CD37, CD81, and CD82 tetraspanins [20,50,62]. Exosomes are usually identified through Western blot or flow cytometry via their most common markers, Alix and CD9, CD63, CD81, and CD82 tetraspanins [26]. Moreover, they also contain a wide range of origin-independent proteins, including lipid rafts proteins,’ conserved’ proteins such as heat shock proteins (HSC70), cytoskeleton proteins (β-actin, myosin, cofilin, and tubulins), metabolic proteins (GADPH and ENO1), major histocompatibility complex (MHC) class I and II, as antigen-presenting vesicles, and cell-type specific proteins, which depend on physiological or pathophysiological conditions. Exosomes also carry cell signalling proteins, such as β-catenin, Delta-like 4, Wnt5B or the Notch ligand, and mediators like interleukin-1β, TNF-α, or TGF-β [27,34,63]. 

Exosomes are also rich in nucleic acid cargo. Micro RNAs (miRNAs), mRNA, other small non-coding RNAs (tRNA, siRNA), long non-coding RNAs, and DNA fragments transfer genetic information to recipient cells [32,64]. It has been demonstrated that miRNA can be selectively sorted into exosomes by interacting with specific RNA-binding proteins [65]. After delivery into the target cells, exosomal miRNAs and mRNAs can regulate gene expression and be translated into new proteins [66]. For example, exosomal miRNAs from T-cells and Epstein virus-infected B-cells can affect the gene expression of dendritic cells (DCs) and monocytes, respectively [67,68]. Neural cells and myoblasts exosomes contain mitochondrial DNA (mtDNA), which might be carried into target cells’ mitochondria, but its role is unclear [69,70].

Several studies corroborate that exosomal miRNAs have a function in tumour microenvironment modulation and are involved in various pathological pathways, including tumorigenesis, invasion, progression, angiogenesis, metastasis, chemo, and radioresistance, and the RIBE induction via the regulation of gene expression [23,44,68,71,72,73]. Thus, exosomal miRNAs could be potential targets in diagnostic and therapeutic applications. 

The exosome lipid composition has been investigated in vesicles from hematopoietic cells, melanoma cells, and oligodendrocytes [27,74]. Typically presenting origin-independent lipid properties, exosomes are usually enriched in cholesterol, sphingolipids, ceramide, and glycerophospholipids with long and saturated fatty-acyl chains, as well as lipid mediators like prostaglandins, phospholipase A2, and a phospholipid scramblase, and phospholipases C and D [75,76].

### 2.2. Intercellular Communication

As key mediators in intercellular communication, exosomes can deliver complex signals that contribute to apoptosis, survival, division, growth, and differentiation in physiological and pathological conditions [20]. Communication occurs via a few mechanisms, including (i) interaction of exosomal surface proteins (tetraspanins, laminin, fibronectin, integrins, and proteoglycans) or lipids (phosphatidylserine) with their corresponding receptors on target cells [77,78], (ii) interaction of the produced soluble fragments through proteolytic cleavage of exosomal membrane proteins (L1 neural adhesion molecule, CD44, and CD46 undergoing A disintegrin and metalloproteinase 10) with their receptors on the cell surface [79,80,81], (iii) exosomal internalisation by membrane fusion or phagocytosis with target cells and releasing their content inside recipient cells and (iv) direct transfer of vesicle cargo via connexin-containing channels formed by the docking between exosomes and target cells [82].

The internalisation mechanism is the most common. It is associated with various passive endocytic mechanisms, including mediated endocytosis through the cytoskeleton, clathrin, caveolin, lipid-raft, phagocytosis, and micropinocytosis [32,83]. Internalisation occurs rapidly; exosomes can be identified in recipient cells 15 min after the presentation [78]. Internalisation mechanisms and efficiency are determined by the cell types involved and pathological conditions [32]. For example, glioblastoma cells’ internalisation of glioblastoma-derived exosomes was significantly higher than the astrocyte-derived ones [84]. Moreover, mantle-cell lymphoma (MCL) derived exosomes showed a high internalisation efficiency by MCL cells regarding bone marrow stroma and T-cell leukaemia cells [85]. In vitro and in vivo studies revealed that minor variations in exosomal compositions (like tetraspanins, integrins, and proteoglycans which have critical roles in selective binding, cell adhesion, and migration) can highly affect their binding target cells [86,87,88]. Following internalisation, exosomes can ultimately affect target cells’ biological functions and behaviour [34], as further discussed in the next section. 

## 3. Exosomes and Disease

Exosomes production, content, and function primarily depend on the parental cells. Nonetheless, external and internal factors, such as disease states, stress conditions, environmental conditions, pharmacological treatments, and ionising radiation, may influence their phenotype [47,89]. 

### 3.1. Disease-Induced Alterations on Exosomes

In the context of cancer, exosomes can regulate adaptive and innate immune responses by immunomodulatory functions. T-cells can be activated by exosomal MHC-peptide complexes binding to their cognate T-cell receptor or exosomal internalisation and processing by APCs [70,81]. Moreover, tumour-derived exosomes can be involved in immunosuppressive response by decreasing T-lymphocytes and natural killer cells proliferation and differentiation and stimulating regulatory T-cells and myeloid cells. Tumour-derived exosomes may also enhance tumorigenesis by secretion of epidermal growth factor receptor (EGFR) ligands like amphiregulin [82]. Additionally, metastasis may be facilitated by providing a favourable extracellular environment for the circulation of tumour cells in chemo/radioresistance and RIBE [15,20,66]. It is also suggested that exosomes spread other pathogens such as viruses or prions between cells [60].

Cancer patients have higher levels of circulating exosomes in the blood, pointing to their putative role as biomarkers [90]. Gai et al. found an expression of miR-302b-3p and miR-517b-3p in exosomes from patients with oral squamous cell carcinoma and an upper-regulation of miR-512-3p and miR-412-3p in salivary extracellular vesicles [91]. Also, the analysis of urinary exosomes from prostate cancer patients showed significant downregulation of miR-375 and up-regulation of miR-451a, miR-486-3p, and miR-486-5p that could be correlated to T stage and bone metastasis of these patients [92].

### 3.2. Cancer Therapeutics and Exosomes

The treatment of human diseases, including chemotherapeutics used in cancer, induces exosome biogenesis, release, and function alterations. Cisplatin-treated ovarian cancer cell exosomes increase invasion and resistance through bystander effects [93]. Likewise, the release of exosomes is involved in the oral squamous cell carcinoma cisplatin resistance mechanism via miR-155 [94]. Otherwise, upregulated exosomal miR-193a derived from bone marrow mesenchymal stem cells leads to suppression of colony formation, invasion, and proliferation as well as improvement apoptosis in cisplatin-resistant cells of non-small cell lung cancer [95].

### 3.3. Other Factors Influencing Exosomes

Hypoxia is an important modulator of exosome production. The secretion of exomes by hypoxic cells and their role in communication between tumoral and stromal cells, within the tumor niche, have been hypothesized to promote the adaptation of cancer cells to hypoxia and tumor growth [96]. These cellular adaptations are mediated mainly by hypoxia-inducible factors (HIFs), a family of transcription factors that, under normoxic conditions, are continuously degraded. However, under hypoxic conditions, there is an accumulation of HIF proteins responsible for activating signalling pathways where exosomes play a relevant role. Moreover, hypoxia also creates an acidic microenvironment, a factor that also increases exosome release [97]. Thus, generally, hypoxic conditions lead to increased exosome secretion. Exosome cargo could be altered by the oxygenation status [47]. Radiation and hypoxia enhanced the effect of exosome-induced metastasis on recipient lung cancer cells and promoted angiogenesis on endothelial cells [98]. Hypoxic glioblastoma exosomes have greater ex vivo and in vitro angiogenic effects than normoxic ones, by stimulating hypoxic cells to secrete more growth factors and cytokines [99]. This exosome-mediated adaptive cellular response to hypoxia could enhance the radioresistance of cancer cells by multiple cellular responses and, consequently, negatively influence radiotherapy outcomes [96,100]. 

## 4. Radiation Influence on Exosome Composition and Function

Exosomes induced by IR and their cargo are influenced by the type of radiation, time, dose, and cell type exposed. The exosome release seems to be dose-dependent, with an increase in their secretion with increasing doses of X-ray [96]. Moreover, it was found that high LET radiation induced more damage in bystander cells, with an increased number of micronuclei formation compared to low LET radiation. These effects are associated with an increase in ROS production [9]. Furthermore, high LET (Carbon ions) radiation induced more secretion of bystander factors in chondrocytes, compared to low LET (X-ray). These factors lead to a decrease in cell survival and proliferation as well as increased DNA damage. TNF-α and IL-6 factors were involved in mediating these RIBE effects, with the highest levels of these factors being detected after high LET irradiation [101]. Is still not well known the real role of exosomes in mediating these RIBE effects, particularly for high LET radiation [102]. 

Many studies, such as those presented in Table 1, on normal and tumour cell lines, report that radiation impacts exosome-based intercellular communication. Cells exposed to ionising radiation (IR) increase exosome release [22,48,103] and influence the composition of exosomal proteins involved in transcription, translocation, and cell division [104]. These small vesicles can influence near and distant tissues by mediating DNA damage and genomic instability [29,105].

Lehmann et al. reported that exosomal CD276 of the 22RV1 prostate cancer cell line was augmented following IR exposure, associated with an increase in premature cellular senescence [106]. Exosomal survivin was related to cancer recurrence after RT since HeLa cells treated with a sublethal dose of proton irradiation demonstrated an enhancement of this exosomal protein [109]. 

Radicals produced by IR break chemical bonds and oxidise the adjacent molecules. Consequently, there is an increase in reactive oxygen species (ROS), nitric oxide (NO), cytokines, DNA damage, and disturbance in calcium transport. DNA damage-activated p53 transcription factors stimulate the formation and release of exosomes [110]. 

Dinh et al. proposed, for the first time, circulating exosomal miRNAs as RT toxicity prediction tools. They verified that radiation dosage reduced miR29a-3p and miR150-5p expression in exosomes derived from advanced non-small cell lung cancer (NSCLC) patients [111]. Studies on prostate cancer cells showed that IR was accompanied by an increased CD276 and Hsp72 [106,112]. In the case of glioblastoma cell lines, an elevated level of connective tissue growth factor (CTGF) and insulin-like growth factor-binding protein 2 (IGFBP2) have been reported [22]. An analysis of exosome components released by an IR exposed human squamous head and neck cell line (FaDu) demonstrated a significant change in the exosomal cargo with an elevated cellular level of transcription, translation, cell division, and cell signalling protein species [105].

Exosome migration and internalisation could increase the chance of exosomal cargo from radiation-targeted cells reaching the distant cells and RIBE in the non-targeted cells. A study on MCF7 cells revealed the synergic effect of protein and RNA exosome components to induce RIBE and spread genetic instability and inflammation in the neighbouring cells [48]. The cellular uptake of exosomes increased through CD29/CD81 complex formation after human bone marrow-derived MSC were exposed to ionising radiation [113]. These results indicate that both exosome release from exposed cells and uptake by recipient cells could be affected by ionising radiation. Another example of the intercellular communication affected by IR is the enhancement of exosome recipient cells migration and invasion in glioblastoma cell lines due to elevated activation of TrkA and FAK signalling [22]. Incorporating radiation-induced exosomal cargo was associated with promoted tumour cell motility and pre-metastatic niche formation during RT. A proteomics study on exosomes derived from irradiated HNSCC cells showed AKT signalling increase, thereby imparting a migratory phenotype in recipient cells, resulting in HNSCC progression during radiotherapy [107]. 

As a critical feature of the tumour microenvironment, exosomes have an active role in developing radioresistance. The serum miRNA profiles of lung cancer patients were evaluated after radiotherapy. miR-208a developed proliferation and radioresistance in lung cancer cells by targeting p21 and activating AKT/mTOR pathway [108]. Exosomes are involved in cancer radioresistance through exosomal non-coding RNAs, mRNAs, and signalling pathways. Still, tumour type and tumour microenvironment interfere with exosomes in cancer radioresistance [114,115]. 

## 5. Exosomes in the Bystander Effect

For a long time, conventional radiobiology stated that biological effects of radiation are associated with irreparable or misrepaired DNA damages (single or double-strand breaks, crosslinks of DNA-DNA and -protein, and chromosome aberrations) in cells directly irradiated. The radiation-induced DNA damages result from direct energy deposition into major cellular structures, such as DNA, or indirect damage via produced reactive oxygen species (ROS) through radiolysis of water molecules. These are described as radiation-induced targeted effects [32,116]. However, this concept has changed with a growing number of studies where irradiation damage was observed not only on targeted cells but also on nonirradiated or distant cells, which exhibit genetic mutations, chromosome damage, micronuclei, and apoptosis, as a consequence of intercellular communications, secreted molecules or signals from directly irradiated cells [11,117,118]. These phenomena are referred to as radiation-induced nontargeted effects, also termed at that time as RIBE. As aforementioned, three types of nontargeted effects are known, and RIBEs are the bystander effects [119]. RIBEs were first demonstrated by Nagasawa et al. in 1992 via investigation of induced damages by a low dose of α-particles. As a result, chromosomal damage was seen in more than 30% of a cell population, wherein only less than 1% of cells had the cell nuclei targeted by the particles. Then, RIBEs were so far corroborated by many in vivo and in vitro studies [8,120,121].

RIBE can potentially lead to biological damage to normal tissues, with the risk of radiation-induced secondary tumours. Late distant secondary tumours in patients who underwent radiotherapy are concerned with the growing prevalence and mortality rate of cancer. Thus, RIBE mechanisms gained considerable attention, aiming for radiotherapeutic strategies with an effective response and low risk of radiation-induced secondary tumours [21,122,123].

The mechanisms involved in bystander effects are complex, and the detailed molecular mechanism is unclear [124]. Intercellular communications between irradiated and nonirradiated cells can be mediated by the delivery of soluble signalling factors secreted from irradiated cells, which depends on the cell type and its state, including cytokines such as interleukins, TNF-α, TGF-β, miRNA, ROS, and nitric oxide. Calcium fluxes to distanced nonirradiated cells or through intercellular gap junctions from the irradiated cells to the neighbouring nonirradiated cells [32]. More recently, the involvement of exosomes secreted from irradiated cells or bystander cells has been identified in the systemic response to radiation [18,48,125]. Thus, exosomes released by neighbouring nonirradiated cells, i.e., bystander cells, can initiate multiple signalling pathways and mediate short or distant non-targeted effect effects.

RIBE-induced exosomes can be initiated by radiation-induced DNA damages and activation of TSAP6 protein and P53 transcription factor-related pathways [17,110]. Exosomes may constitute a vehicle for radiation-induced signalling factors, protected from degradation by extracellular enzymes and ultimately delivered to distanced nonirradiated cells. Recent studies on RIBE-mediated exosomes are summarised in Table 2. It is reported that exosomal proteins and mRNA are key signalling factors prompting RIBE in the recipient cells [48,72,125].

The exosomes induced by RIBE, and their associated effects, could depend on the type of radiation. It has been hypothesized that high-LET radiation could increase exosome release considering their relevant role in non-targeted effects but their different response is still to be clearly elucidated [126]. A recent study showed an increased level of exomes release after human bronchial epithelial cells irradiation with 1 Gy of high charge and energy (HZE) ions than with 3 or 10 Gy of ɣ-ray. These exosomes are enriched in molecular patterns related to pro-inflammatory damage, such as HSP70 and calreticulin [127].

**Table 2:** RIBE effects mediated by exosomes released by various irradiated or nonirradiated bystander cells or organs.

Irradiated Cells or Organ	Dose	Nonirradiated Bystander Cells or Organ	RIBE-Induced Exosomes	Reference
Focal brain of C57BL/6 and LC3B-GFP transgenic mice	10 Gy (X-ray)	lung tissues	Significant increase of the miR-7 expression in astrocytes and oligodendrocytes. Significant increase of LC3B, LC3B-GFP, Beclin-1, and miR-7 levels in lung cells after irradiation. miR-7 mediated autophagy in distant lung tissues. Significant decrease of Bcl-2 levels (direct target gene of miR-7) in lung cells after brain irradiation.	[72]
Seven-week-old male ICR mice and normal human dermal fibroblast (HDFn) cells	4 Gy (X-ray)	mouse embryonic fibroblast (m5S) cells and human fibroblast cells (HDFn cells)	Significant increase of mitochondrial DNA (mtDNA) in derived exosomes from 4Gy irradiated mouse serum and HDFn cells. Induction of DNA damage and RIBE signals in bystander cells mediated by mtDNA.	[128]
SH-SY5Y and SK-N-BE human neuroblastoma cell lines	0.1, 1, 5, and 10 Gy (X-ray)	SH-SY5Y cells	Significant increase in viability of nonirradiated recipient cells. Stimulation of proliferation and cell survival. Increase cell migration via AKT activation. Increase in the rate of DNA break repair.	[129]
C57BL/6 mice	2 Gy (X-ray)	Intravenous injection of isolated exosomes from the bone marrow into unirradiated (so-called bystander) animals	Induction of γ-H2AX foci formation in the spleen of recipient mice. miRNAs mediated the increase of chromosomal aberrations and the activation of the DNA damage response in EV-recipient. Induction of quantitative changes in the cellular composition of bone marrow and spleen of recipient mice.	[130,131]
C57BL/6 mice	0.1, 0.25, and 2 Gy (X-ray)	Intravenous injection of isolated exosomes from bone marrow 4, 24 h, and 3 months after irradiation into unirradiated (so-called bystander) animals	Systemic increase in the circulating reactive oxygen metabolite levels and a reduced expression of antioxidant enzyme genes and iNOS2 in bystander mice. The cell number decrease and the increase in cellular apoptosis observed in hematopoietic cells of bystander animals were similar to the effects observed in irradiated mice. These effects were persistent for up to 3 months.	[132,133]
Abl-µNLS mouse embryo fibroblasts	10 Gy (ɣ-ray)	Abl-WT mouse embryo fibroblasts	Inhibition of colony formation in unirradiated cells by increasing reactive oxygen species (ROS). Increase of miR-34c levels.	[134]
SH-SY5Y human neuroblastoma cells, U87 glioma cells, and STS26T human malignant peripheral nerve sheath tumour cells	3, 12 Gy (Source not disclosed)	SH-SY5Y human neuroblastoma cells, U87 glioma cells, and STS26T human malignant peripheral nerve sheath tumour cells/U87-nude mice	Significant increase in cell proliferation and survival. Decrease in ROS production. Enhancement of tumour burden in the mice and decrease in survival.	[135]
neonatal mice and exosomes secreted from cheek skin tissues and back skin tissues	7 Gy (X-ray)	m5S and MEF mouse fibroblast cell lines	Reduced colony-forming efficiency in bystander cells. Radiation-protective effects of derived exosomes from cheek skin tissues on irradiated m5S and MEF cells. Faster repair of DNA double-strand breaks in m5S and MEF cells treated with derived exosomes from cheek skin tissues.	[136]
human bronchial epithelial cells (HBEC3-KT F25F cells)	1 Gy of ^48^Ti, ^28^Si, or ^16^O (HZE ions) 3 Gy or 10 Gy (ɣ-ray)	HBEC3-KT F25F cell	Exosome released after high-LET irradiation with HZE ions is about 4-fold with HZE ions compared to control. Pro-inflammatory damage and associated patterns, such as HSP70 and calreticulin, were detected in exosome-enriched vesicles preparations.	[127]

**Figure 3:**
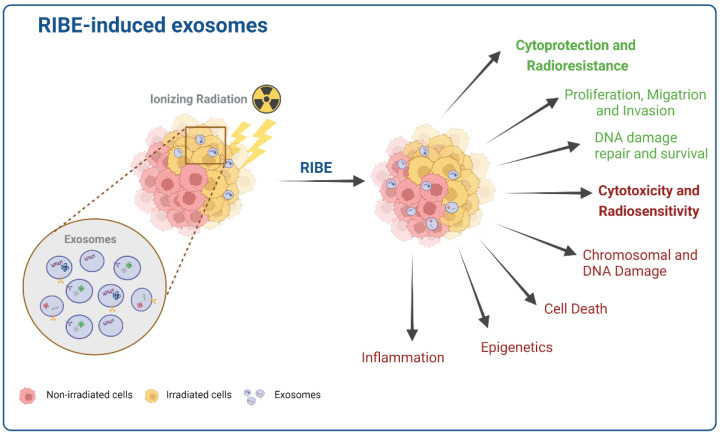
Exosomes-induced RIBE in recipient cells enhances the mechanism associated with cytoprotecting and cancer radioresistance (green) or, on the opposite, the mechanism associated with cancer radiosensitivity and cytotoxicity (red). Created with BioRender.com.

It is proposed that exosomes released by irradiated or bystander cells could lead to two opposite functions in recipient cells. First is a potentially cytoprotective function, promoting migration and metastasis and enhancing DNA damage repair and cell survival. Second, a potentially cytotoxic function where inflammation, chromosomal damage, epigenetics, and ultimately cell death occur [8,48,73,107,125]. The relevant mechanistic diagram is shown in Figure 3.

In the case of the cytoprotective function, exosomes of a glioblastoma cell line irradiated with a 4 Gy dose lead to enhanced migratory activity in nonirradiated recipient cells [22]. Exosomes are involved in the cell migration/invasion signaling and can enhance the other molecule’s activity in these signaling pathways, including neurotrophic tyrosine kinase receptor type 1 (TrkA), focal adhesion kinase, Paxillin, and proto-oncogene tyrosine-protein kinase Src (Src) in recipient cells [22].

In accordance, Mutschelknaus and coworkers showed that exosomes isolated from 6 Gy irradiated cells increased the motility and migration of the recipient HNSCC cells (BHY and FaDu). AKT-signalling was a vital regulator in mediation exosome-induced migration, with an increased signaling after irradiation [107,137]. Molecular findings denoted increased phospho-mTOR, phospho-rpS6, and MMP2/9 protease activity. Thus, exosomes could promote activation of the AKT signalling in recipient cells. Moreover, BHY cells’ exosome proteome showed IR upregulated 39 proteins and downregulated 36. For example, FGFR1, HSP90AA1, HSP90AB1, HSP90B1, and VTN were upregulated and can be involved in AKT activation, MMP243 stability, enhancement of exosome-mediated motility, and metastasis in recipient cells [107].

As stated, irradiated or bystander cells exosomes could have a cytoprotective role in recipient cells through increasing cell survival and regulation of DNA repair. The proteins involved in DNA damage repair are overrepresented exosomes cargo. The proteomic profile of 2 Gy irradiated FaDu cells exosomes presented an increased expression of transcription and translation proteins, chaperones, ubiquitination-related factors, and proteasome components [104]. UM-SCC6 cells showed a dose-dependent proteome with 472 total proteins, including 425 upregulated and 47 downregulated. Among the overrepresented, were found proteins involved in response to radiation, metabolism of radical oxygen species, DNA repair, chromatin packaging, and protein folding [125]. Interestingly, irradiated cells’ exosome uptake is superior to nonirradiated BHY and FaDu cells [138].

Regarding the cytotoxic function of exosomes derived from irradiated cells, apoptotic and stress signalling factors are overrepresented. These could mediate RIBE resulting in inflammation and chromosomal damages in the nearby nonirradiated cells [125].

Jella et al. investigated exosome involvement in the RIBE process in nonirradiated human keratinocytes cells. They demonstrated that exosomes derived from HaCaT keratinocytes promote calcium influx, ROS production, and cell death in nonirradiated cells [103]. Exosomes released from irradiated MCF-7 breast cancer cells induced specific RIBE responses in nonirradiated MCF-7 breast cancer cells [139]. Interestingly, the longevity of exosome RIBE-inducing activity in the progeny of irradiated and bystander cells was also evaluated, and the role of certain exosomes’ RNA and proteins [48]. Exosomal proteins released directly by irradiated cells or secreted from bystander cells, and their progeny, can mediate intracellular communication for inflammatory response [140].

Recent research revealed that exosomal-mediated miRNAs, as messengers between irradiated and nonirradiated bystander cells, have an important role in initiating RIBE. miRNAs are an important class of non-coding RNAs regulating gene expression and were investigated as potential RIBE mediators [8,141,142]. A range of studies has shown that the indirect involvement of miRNAs can epigenetically regulate delayed RIBE. Dickey et al. reviewed the role of miRNA in the indirect effects of ionising radiation. They suggested that miRNAs can be considered non-primary bystander signals promotors of DNA double-strand breaks and apoptosis [141]. Moreover, some studies have followed and validated the role of miRNAs in the induction of delayed RIBE in the different human tissue and animal models through the expression changes of BCL-2, DNA methylation, and ultimately apoptosis [48,139,143,144].

miR-21 is a well-described DDR (DNA damage response)-related miRNA, which was investigated on unirradiated WS1 human fibroblasts cells after co-culture with α-irradiated HaCaT keratinocytes cells. The expression of miR-21, ROS levels, and p53 binding protein 1 (53BP1) foci (a double-strand break (DSB) marker) significantly increased in unirradiated bystander WS1 cells [145,146]. Other studies demonstrated that miR-21 participated in RIBE [143,147].

An increased frequency of micronuclei and the 53BP1 foci in the nonirradiated MRC-5 cells indicated that exosomes from 2 Gy irradiated MRC5 cells could induce DNA damage in the bystander cells. Furthermore, nonirradiated MRC5 cells treated with miR-21-containing exosomes showed a significant expression of miR-21 and a remarkable suppression in the level of Bcl2 (as a target gene of miR-21). This exosomal cargo can induce chromosome aberration and DNA damage in nonirradiated bystander MRC5 cells by targeting Bcl2 [147].

Exosomes secreted by 2 Gy irradiated BEP2D cells contain increased miR1246. Inhibition of proliferation and induction of DNA damage via downregulation of the DNA Ligase4 (LIG4) gene expression by directly targeting its 3′UTR was observed in nonirradiated cells incubated with the vesicles [73].

Proteome analysis performed on isolated exosomes from 2 Gy irradiated blood samples from healthy individuals showed downregulation of afamin and serpine peptidase F1 and overexpression in miRNAs of miR-204-5p, miR-92a-3p, and miR-31-5p [148]. These can be important messengers in RIBE induction and regulation.

Exosomes-containing miR-7-5p contributed to crucial endpoints of RIBEs like autophagy in nonirradiated cells [8]. This study identified a range of upregulated miRNAs in the exosomes secreted by 2 Gy irradiated human bronchial epithelial BEP2D cells. Among these upregulated exosomal miRNAs, miR-7-5p induced autophagy in nonirradiated cells, and the miR-7-5p inhibitor remarkably decreased this bystander effect [8].

It is also essential to consider the methodologies and techniques used in the analysis of exosomes, considering that there are few standard guidelines for the exosomes research field [149]. The isolation and purification processes are mainly conducted by ultracentrifugation. Still, gradient-based and size-exclusion chromatography isolation methods could be used, resulting in the purification of vesicles with different concentrations, purity, and size [47]. Cell culture medium and conditions are also crucial, such as glucose status, antibiotics, and enriched protein supplements (foetal bovine serum). Additionally, it is observed an increase in the exosome release under acidic conditions (pH 6.5) compared to physiological (pH 7.4) [47,150]. These factors should be considered while analysing the results obtained.

## 6. Conclusions

RIBEs are a set of induced biological effects in nonirradiated cells via cellular communications, regarded as a prominent issue in the radiotherapy protocol. Bystander effects lead to potential hazards of nonirradiated normal cells and ultimately the development of secondary cancers. These effects can also be useful to kill nonirradiated cancer cells during radiotherapy. Therefore, the involved intracellular communications in RIBE can be used as specific targets in designing and developing new radiosensitisers and inhibitors to upregulate and reduce the RIBEs, respectively.

The mechanisms involved in bystander effects are complicated, and the detailed molecular mechanisms are not well comprehended. Recent studies indicate that exosomes mediate intercellular communications between irradiated and nonirradiated cells. Exosome-based RIBE can be stimulated by radiation-induced DNA damage and activation of TSAP6 protein and P53 transcription factor-related pathways. Radiation could also be associated with changes in the secretion profile and the composition of exosomes. The specific molecular cargo of exosomes, proteins, and RNA, have a crucial role in transferring signals and prompting RIBE in the neighbouring nonirradiated cells, i.e., bystander cells. Then, the exosome can initiate multiple intracellular signalling pathways and mediate short or distant non-targeted effects. Intracellular communication of the released exosomes can occur through several mechanisms, being internalisation the most common. The studies demonstrate that exosomes released by irradiated cells could lead to two opposite functions in the nonirradiated cells: (i) cytoprotective function, such as promoting migration and metastasis and enhancing DNA damage repair and cell survival, and; (ii) cytotoxic function associated with inflammation, chromosomal damage, epigenetics and ultimately cell death.

The data illustrate the importance of exosomes in RIBE and their contribution to general radiation-induced non-targeted effects and potential as targets in therapeutic protocols. However, it is important to highlight that the currently available data is based on in vitro and in vivo research. Therefore, further studies are required to establish the role of exosomes in RIBEs and explore the inherent molecular mechanisms involved in exosomes release and cargo, especially depending on the type of radiation. Combining this information with other functions of exosomes in non-targeted effects, such as abscopal and cohort, may allow moving forward into new efficient clinical approaches for radiotherapy-based cancer treatment.

## Figures and Tables

**Table 1:** Influence of the ionising radiation on exosomes’ characteristics from different cell lines.

Cell Line	Dose	Radiation	Results	Reference
Human epithelial prostate cell carcinoma (22Rv1)	4 Gy	ɣ-ray	Increased release of exosomal CD276.	[106]
Human head and neck squamous cell carcinoma (FaDu)	2 Gy	X-ray	Elevated levels of transcription, translation, cell division, and cell signalling factors.	[104]
Human glioblastoma multiforme (U87MG)	2, 4, 6, 8 Gy	X-ray	Elevated TrkA and FAK signalling; enhancement of the recipient cells migration.	[22]
Human head and neck squamous cell carcinoma (FaDu, BHY)	6 Gy	ɣ-ray	Pro-migratory phenotype induction; enhanced HNSCC progression.	[107]
Human NSCLC cell lines (A549, H1299, H1975, and H460)	2, 4, 6, 8 Gy	X-ray	Activation of AKT/mTOR pathway; radioresistance.	[108]

## Data Availability

Data sharing not applicable.
